# Gold nanorods/tetrahedral DNA composites for chemo-photothermal therapy

**DOI:** 10.1093/rb/rbac032

**Published:** 2022-05-04

**Authors:** Ziyun He, Qiusheng Wang, Nan Zhang, Jianqin Yan, Li Li, Jun Cao, Bin He

**Affiliations:** 1 National Engineering Research Center for Biomaterials, College of Biomedical Engineering, Sichuan University, Chengdu 610064, China; 2 Department of Pharmaceutics, School of Pharmacy, Qingdao University, Qingdao 266073, China

**Keywords:** nanocomposite, DNA tetrahedron, gold nanorod, chemo-photothermal therapy

## Abstract

Combination therapy is extensively developed for cancer treatment in recent years due to its high efficiency. Herein, we constructed a nanocomposite based on gold nanorods (GNRs) and drug-loaded tetrahedral DNA nanostructures (TDN) for chemo-photothermal combinational therapy. Anti-tumor drug doxorubicin (DOX) was loaded via the insertion within GC base pairs of TDN. The aptamer AS1411 was attached to the apex of TDN (ATDN) to target tumor cells. The DOX-loaded DNA tetrahedron (ATDN-DOX) was compressed by the GNRs coated with PEI (GNRs@ATDN-DOX) to realize the photothermal function and lysosome escape. GNRs under the illumination of 808 nm infrared laser showed high photothermal conversion and stability due to the protection of PEI layer. The drug-loading capacity of ATDN-DOX was as high as 314 DOX molecules in per ATDN. The positive charge of PEI in GNRs@ATDN-DOX nanocomposites was utilized to achieve excellent cell penetration and induce proton sponge effect for lysosomal escape. The nanocomposites presented HeLa and 4T1 cells targeting and resulted in efficient anticancer activity.

## Graphical Abstract

**Figure:**
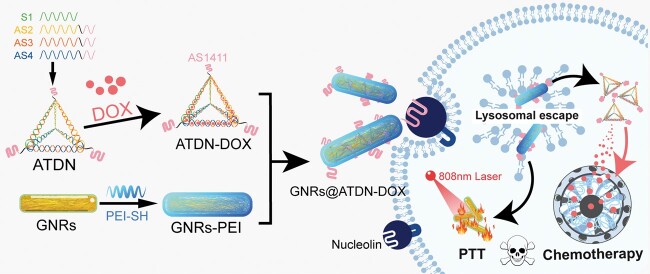

## Introduction

Cancers have emerged as one of the major worldwide health problems [1–3] to cause death over the past few decades [4]. The treatments including radiotherapy [5], surgery [6] and chemotherapy [7] were suffered substantial problems such as the risk of recurrence and metastasis due to incomplete resection [8], and serious side effects [9, 10]. Nanomedicines [11–13] were developed to overcome these problems, nanoparticles of cationic polymers [14], liposomes [15], carbon nanotubes [16], metal–organic frameworks [17] and gold nanorods (GNRs) [18] were reported to load and deliver anticancer drugs. Tetrahedral DNA nanostructures (TDN), a 3D wireframe nanostructure assembled by four oligonucleotides through the base complementary pairing principle [19], have demonstrated as an excellent nanovehicle with high drug-loading, sustaining release capability, easily fabricating for pre-designed sizes and shapes [20] and convenient functionalization [21].

Anticancer drug doxorubicin (DOX) inhibits topoisomerase II within the nucleus and intercalates into DNA to induce cell apoptosis [22, 23], it is widely used in treating solid tumors and hematological malignancies [24]. However, the short half-life, severe drug tolerance and serious cardiotoxicity of DOX are the disadvantages [25]. DOX was reported to intercalate noncovalently into GC base pairs of TDN [26], but the delivery efficiency was poor due to lack of tumor-targeting ability [27]. AS1411 aptamer, an anti-proliferative oligonucleotide, is utilized as a tumor-targeting agent by binding with nucleolin [28], which is a nucleolus protein overexpressed at plasma membrane and cytoplasm of cancer cells (such as breast cancer and melanoma) [29]. TDN immobilized with AS1411 could target tumor cells to enhance delivery efficacy.

Nowadays, more effective therapeutic models including photothermal therapy (PTT) [30], photodynamic therapy [31, 32], immunotherapy [33] and gene therapy [34] have been exploited to avoid drawbacks of traditional methods. With the merits of low invasiveness, user-friendly control, effective targeting, low toxicity and controllable dose, PTT has received much attention [35, 36]. Photothermal agents kill cancer cells with rapid increased surrounding environmental temperature via harvesting near-infrared (NIR) laser energy [37]. GNRs with particular longitudinal surface plasmon resonances (LSPR) [38], excellent photothermal conversion ability [39, 40] and controllable size [41] have been widely applied in tumor treatment [42]. The functionalization of GNRs with gold–thiol bond chemistry [43], layer-by-layer deposition [44] and round-trip phase transfer ligand exchange provided the possibility of drug loading and avoided the strong cytotoxicity caused by cetyltrimethylammonium bromide (CTAB) used in the GNRs preparation [45].

**Figure 1.**
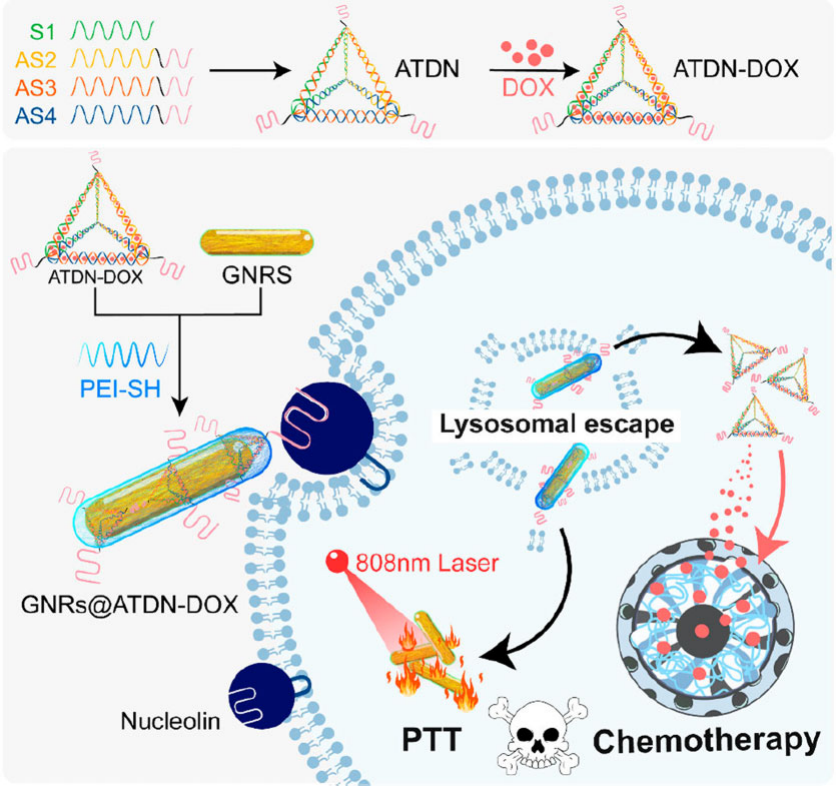
The fabrication of gold nanorods/tetrahedral DNA nanocomposites and the mechanism of chemo-photothermal therapy

Mono-therapy has major limitations due to the intrinsic heterogeneity and complexity of tumors [46]. It is pressing to create multifunctional nano-treatment platform integrated with several effective anticancer modalities [47–49]. Herein, we synthesized GNRs through a seed-mediated growth method [50]; thiolated polyethyleneimine (PEI) was applied to modify the surface of GNRs via Au–S bond to enhance cellular uptake and lysosome escape. DOX was loaded in tetrahedral DNA nanostructures (TDN) immobilized with aptamer AS1411 to prepare ATDN-DOX [51]. The PEI-coated GNRs (GNRs-PEI) were compacted with ADN-DOX to construct nanocomposites (GNRs@ATDN-DOX) for chemo-PTT (Figure 1).

## Experimental section

### Materials

Branched polyethyleneimine (*M*_w_: 25 kDa) and 4-dimethylaminopyridine (DMAP) were acquired from Sigma-Aldrich Co. (Steinheim, Germany). *N*-hydroxysuccinimide (NHS) was provided by Astatech (Chengdu, China). 1-(3-Dimethylaminopropyl)-3-ethylcarbodiimide (EDC) was received from Heowns (Tianjin, China). All oligonucleotides (the nucleotide sequences as shown in Supplementary Table S1) were synthesized by Sangon Biotech Co., Ltd. (Shanghai, China). Doxorubicin hydrochloride (DOX·HCl) was obtained from Aladdin (Shanghai, China).

### Synthesis of PEI-LA

Lipoic acid (LA; 4.3 mg), 2.4 mg of NHS and 3.88 mg of EDC were dissolved in 5 ml of DMSO at 25 °C with stirring for 2 h under the argon atmosphere; 10 ml of DMSO containing 1.25 g of PEI and 0.105 mg of DMAP were mixed with LA solution and continued agitation for 24 h. The product was separated via centrifuging precipitate in acetonitrile. PEI-LA was purified by dialysis (the MWCO of 1000 kDa) and collected via freeze-drying. The product was characterized by ^1^hydrogen-nuclear magnetic resonance and Fourier Transform Infrared Spectroscopy (FTIR).

### Synthesis of GNRs-PEI

The GNRs were prepared through a seed-mediated growth strategy as our previous work [50]. The concentration of GNRs was determined by the UV absorbance at 800 nm (ε = 4.675 × 10^9^ L mol^−1 ^cm^−1^). GNRs-PEI was prepared with bonding thiolated PEI on the outer sphere of GNRs by Au–S bond. The excess CTAB on GNRs was removed by centrifugation at 12 000 rpm for 10 min and suspended twice. The mixture contained equal volume of GNRs, and PEI-LA (30 mg ml^−1^) was put into –196 °C, –20 °C, –4 °C and 37 °C for 30 min, respectively. The reactant was thawed at room temperature, unreacted PEI-LA was removed via centrifugation (12 000 rpm, 10 min). The product was centrifuged and resuspended twice and reserved in deionized water to obtain pure GNRs-PEI dispersions. The GNRs-PEI was characterized by transmission electron microscopy (TEM), thermal gravimetric analysis (TGA) and UV–vis absorption spectra.

### Characterization of photothermal response

Dispersions of GNRs-PEI (0.625 nM) in deionized water were employed to study the temperature variation curve of GNRs by 808 nm laser illumination (1 or 2 W cm^−2^). FLIR Thermal Imager recorded the temperature–time curve. The stability of GNRs in cycle photothermal experiments was reflected by the UV–vis absorption curves with and without irradiation.

### Synthesis of TDN and ATDN

TDN was prepared by the assembly of four oligonucleotides through the base complementary pairing principle. Four oligonucleotides (S1, S2, S3 and S4) at the same concentration of 4 μM were dissolved in a TM buffer (20 mM Tris HCl, 5 mM MgCl_2_, pH 8.0). The base complementary pairing of oligonucleotides was taken place at 95 °C for 15 min in metal bath, and then rapidly cooled to 4 °C for 12 h to gain 1 μM of TDN solution. ATDN was prepared with the same procedure using aptamer AS1411-modified oligonucleotides (S1, AS2, AS3 and AS4). TDN and ATDN were dyed with GelRed for 15 min and distinguished by 1% agarose gel electrophoresis (AGE, electrophoresis liquid: 1 × TBE, voltage: 100 V, time: 1.5 h). The morphological characteristics of the TDN and ATDN nanostructures were observed through TEM. The size was measured on a dynamic light scattering (DLS) spectrometer.

### Preparation of ATDN-DOX and GNRs@ATDN-DOX

Equal volume of DOX·HCl (500 μM) and ATDN (1 μM) were mixed and preserved at 37 °C in water bath shaker for 3 h. The extra DOX·HCl was removed by centrifugation at 10 000 rpm for 10 min. The concentration of uncombined DOX in the supernatant was inferred through the standard UV absorption curve of DOX at a wavelength of 483 nm. Then the drug-loading content (DLC) of ATDN-DOX could be easily calculated by the following formula:

$$\mathrm{DLC}\left(\mathrm{wt}\%\right)=\frac{{\mathrm{Mass}}_{\mathrm{Dox}\;\mathrm{loaded}}}{{\mathrm{Mass}}_{\mathrm{ATDN}\;\mathrm{added}}+{\mathrm{Mass}}_{\mathrm{Dox}\;\mathrm{loaded}}}.$$

GNRs@ATDN-DOX was prepared by mixing equal volume of ATDN-DOX (1 μM) and different concentrations of GNRs-PEI at 37 °C for 30 min. The combining abilities of GNRs-PEI and ATDN-DOX with various molar ratios (GNRs/TDN × 10^−3^ = 1.2, 0.6, 0.4, 0.3, 0.15, and 0) were studied by AGE.

### Cell culture and cytotoxicity

The cell cytotoxicity of the blank GNRs, GNRs@ATDN and GNRs@ATDN-DOX nanoparticles against HeLa cervix cancer cells and 4T1 mice breast cancer cells was evaluated by an MTT assay. In short, the HeLa and 4T1 cells were treated with multiple concentrations of GNRs, GNRs@ATDN and GNRs@ATDN-DOX for 4 h. After a few minutes of exposure with NIR laser (808 nm, 1 W cm^−2^), the cells were incubated continually for 20 h. Cells were then cultivated with serum-free medium containing MTT (0.5 mg/ml) for 3 h. After the MTT medium was removed, 100 μL of DMSO was shift-in each well and shaken evenly. The absorbance of formazan was measured at 490 nm using enzyme-linked immunosorbent assay. The cell viability (%) was calculated as follows:

$$\mathrm{Cell}\;\mathrm{Viability}\;\left(\%\right)=\frac{{OD}_{\mathrm{treated}}-{OD}_{\mathrm{MTT}}}{{OD}_{\mathrm{control}}-{OD}_{\mathrm{MTT}}}\times 100\%$$

The cytotoxicity against 3T3 and L929 cells was studied similarly.

For LIVE/DEAD viability/cytotoxicity assay, HeLa and 4T1 cells were seeded in glass-bottom culture dishes at a density of 1 × 10^4^ cells per well and cultivated for 24 h, and then incubated with GNRs@ATDN and GNRs@ATDN-DOX for another 4 h. The samples were treated with 808 nm NIR laser (1 W cm^−2^, 5 min), and then incubated for 20 h. After removing materials in dishes, the cells were incubated with Calcein AM/PI solution for 15 min. The fluorescence of live/dead cells was observed by confocal laser scanning microscope (CLSM, N-SIM/A1R MP+, Nikon, Japan).

### Cellular uptake

To qualitatively analyze the cellular uptake of the nanomedicines, HeLa, 4T1 and 3T3 cells were seeded in 35 mm glass-bottom culture dishes and cultivated for 24 h. The cells were incubated with GNRs@ATDN-DOX and GNRs@TDN-DOX for 4 h, and then fixated with 4% paraformaldehyde at 25 °C for 15 min. Using Hoechst 33342 (10 μg/ml), cells were dyed for 15 min at 37 °C, and excess dyestuff was removed with PBS for three times. The fluorescence intensity of DOX and DAPI was immediately imaged by CLSM.

HeLa, 4T1 and 3T3 cells were cultivated with GNRs@ATDN-DOX and GNRs@TDN-DOX for 4 h. The cells were gathered by digestion and centrifugation (1000 rpm, 5 min) to obtain a cell suspension in PBS. The fluorescence intensity of DOX in cells was counted via a flow cytometer (Becton, Dickinson and Company, USA). Each sample was repeated in triplicate and the number of cells in each sample was not less than 1 × 10^4^.

### Lysosome escape

The lysosome escape of the GNRs-PEI in HeLa and 4T1 cells was described using CLSM. Briefly, HeLa and 4T1 cells were cultured with Cy5-labeled ATDN or GNRs@ATDN for 4 h. Whereafter, the media were removed, and superfluous materials were removed by washing twice with cold PBS. Each well of cells were cultivated with 1 ml of fresh medium at 37 °C for 4 h. Afterwards, two kinds of cells were stained by Lyso-trackers^®^ Green (100 nM) at 37 °C for 40 min and removed redundant dyes with PBS for three times. The lysosome escape ability of the nanoparticles was analyzed via the overlap phenomenon between the fluorescence of Cy5 and Lyso-trackers^®^ Green observed by CLSM.

### Apoptosis assay

Apoptosis of HeLa and 4T1 cells in different stages caused by GNRs@ATDN-DOX were detected by flow cytometer. Dual-staining with APC-conjugated Annexin-V/7-aminoactinomycin D (7-AAD) was exploited using a commercial kit. HeLa and 4T1 cells were disposed with free DOX, GNRs@ATDN and GNRs@ATDN-DOX for 12 h. In the meantime, several groups were irradiated for few minutes via NIR laser (1 W cm^−2^) with 808 nm wavelength. According to the instruction, both treated and control cells were digested with trypsin without EDTA to improve combining capacity of dyes, washed with cold PBS and reserved in 500 μL of Annexin V binding buffer (1×). The cells were treated with 5 μL of Annexin V-APC and 10 μL of 7-AAD solutions in the dark for 10 min. Annexin V-APC and 7-AAD solutions were treated with the corresponding positive, negative or blank control groups, respectively. The samples were analyzed by flow cytometer to evaluate apoptotic and necrotic cells.

## Results and discussion

### Preparation and characterization of GNRs-PEI

As illustrated in Supplementary Fig. S1A, the signal at 2.45–2.79 ppm (c) was attributed to the methylene group on the PEI chain, and multiplet peak at 3.00–3.14 ppm (b) was assigned to the proton in the methylene group near the PEI to the right of the carbonyl group. Additionally, the appearance of the signal at 3.25 ppm (a) belonged to the last methine of LA. FTIR spectra (Supplementary Fig. S1B) demonstrated that the absorbance of carboxyl group at 1691 cm^−1^ was assigned to LA, which disappeared and replaced by amide group at 1643 cm^−1^ and 1569 cm^−1^ in PEI-LA to imply the successful amide reaction.

**Figure 2.**
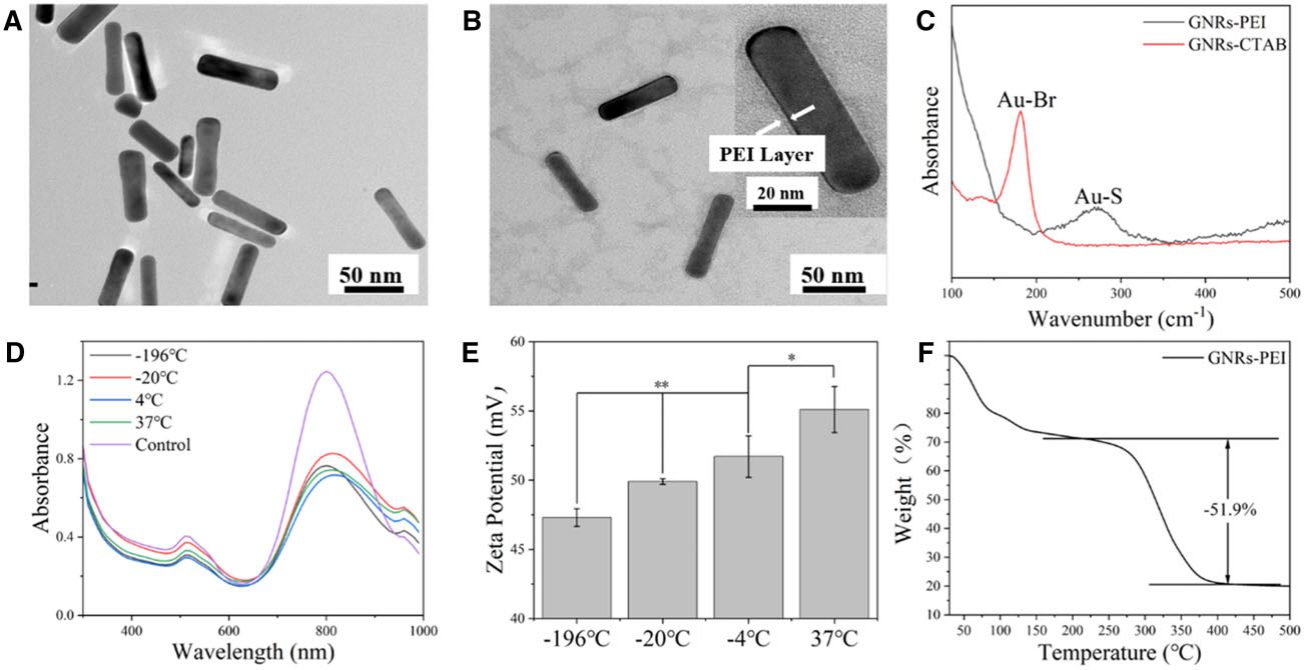
Characterizations of GNRs-PEI. TEM images of GNRs-PEI (**A** and **B**). Raman spectra of GNRs-CTAB and GNRs-PEI (**C**). UV–vis absorption spectra (**D**) and zeta potentials (**E**) of GNRs-PEI at different preparation temperatures. TGA curve of GNRs-PEI (**F**)

TEM images of GNRs-PEI (Fig. 2A and B) showed a limited size distribution with 70 ± 3 nm length, 17 ± 2 nm width and aspect ratio of 4.0 ± 0.3. Raman spectra (Fig. 2C) also revealed that the Au–Br bond at 176 cm^−1^ was replaced by the Au–S bond at 262 cm^−1^. Different from the preparation at –196°C, with the changes of local refractive index caused by more PEI modification under higher temperature, GNRs-PEI prepared at –20, 4 and 37 °C showed a minor redshift of the LSPR band in UV–vis absorption spectra (Fig. 2D). Meanwhile, the zeta potential of GNRs-PEI (Fig. 2E) increased with the increase of preparation temperature, it proved that the amount of PEI in modification was related to temperature. Besides, the decomposition temperature of PEI began around 213 °C and ended around 419 °C in TGA (Fig. 2F). From the calculation, the mass content of PEI in GNRs-PEI was 51.9 wt%. GNRs-CTAB with a similar size (65 ± 3 nm in length, 15 ± 2 nm in width, and 4.0 ± 0.2 in aspect ratio) was synthesized (Supplementary Fig. S3) as a control and its zeta potential was 27.63 ± 1.30 mV.

### Photothermal properties of GNRs-PEI

**Figure 3.**
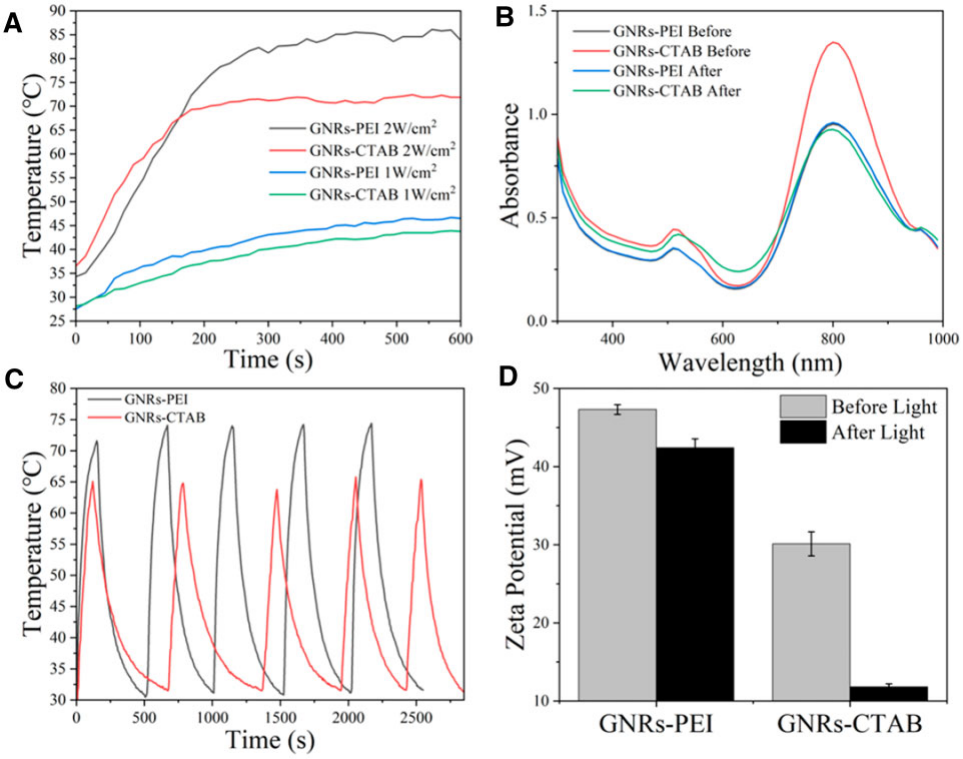
Photothermal stability of GNRs. The heating curve of GNRs illuminated by 808 nm laser with power density of 1 and 2 W cm^−2^ (**A**). UV–vis absorption spectra of GNRs before and after irradiation (**B**). The cycle heating curve of GNRs irradiated by 808 nm laser (2 W cm^−2^) (**C**). Zeta potential of GNRs before and after cycle irradiation (**D**)

The temperature curves of GNRs-PEI dispersions with concentration of 0.625 nM were detected under 808 nm NIR laser (1 and 2 W cm^−2^) exposure (Fig. 3A). When the laser power was 2 W cm^−2^, GNRs displayed a very fast heating rate, exceeding 50 °C in 100 s and 60 °C in 150 s, and the heating rate was gentle after 200 s. The maximum temperature of GNRs-PEI after irradiation could reach 85 °C, while the maximum temperature of GNRs-CTAB after exposure was only 70 °C, illustrating that the PEI coating layer improved the photothermal effect of GNRs. When the laser power was 1 W cm^−2^, the GNRs dispersions displayed a slower heating rate, and the highest temperature after 10-min irradiation was < 45 °C. The highest temperature of GNRs-PEI was slightly higher than that of GNRs-CTAB, the trend was the same as previous ones. As shown in Fig. 3B, the LSPR peak position of GNRs-PEI did not change after irradiation, while blue shift happened on GNRs-CTAB, indicating that the morphological change of GNRs-CTAB occurred after photothermal transformation, which was due to the melting or aggregation of GNRs-CTAB [52].

The photothermal stability of GNRs is important after repeated photothermal conversion as the surface coating of GNRs would fall off to result in agglomeration of GNRs and reduce photothermal effect. The temperature variations of GNRs (0.625 nM) dispersions were detected under 808 nm NIR laser (2 W cm^−2^) exposure for five repeated times (Fig. 3C and D). As a result, the heating rate of GNRs-PEI was nearly unchanged, the highest temperature maintained at 70 °C, and the zeta potential was only slightly reduced after five repeated irradiations, illustrating that the PEI coating layer was not lost, which provided a good protective effect. The highest temperature of GNRs-CTAB was slightly lower than that of GNRs-PEI, it fluctuated around 65 °C, and the zeta potential decreased significantly after the cycle, implying that the CTAB layer was exfoliated during the photothermal process, which resulted in decrease in zeta potential and prone aggregation.

### Characterizations of TDN and ATDN

**Figure 4.**
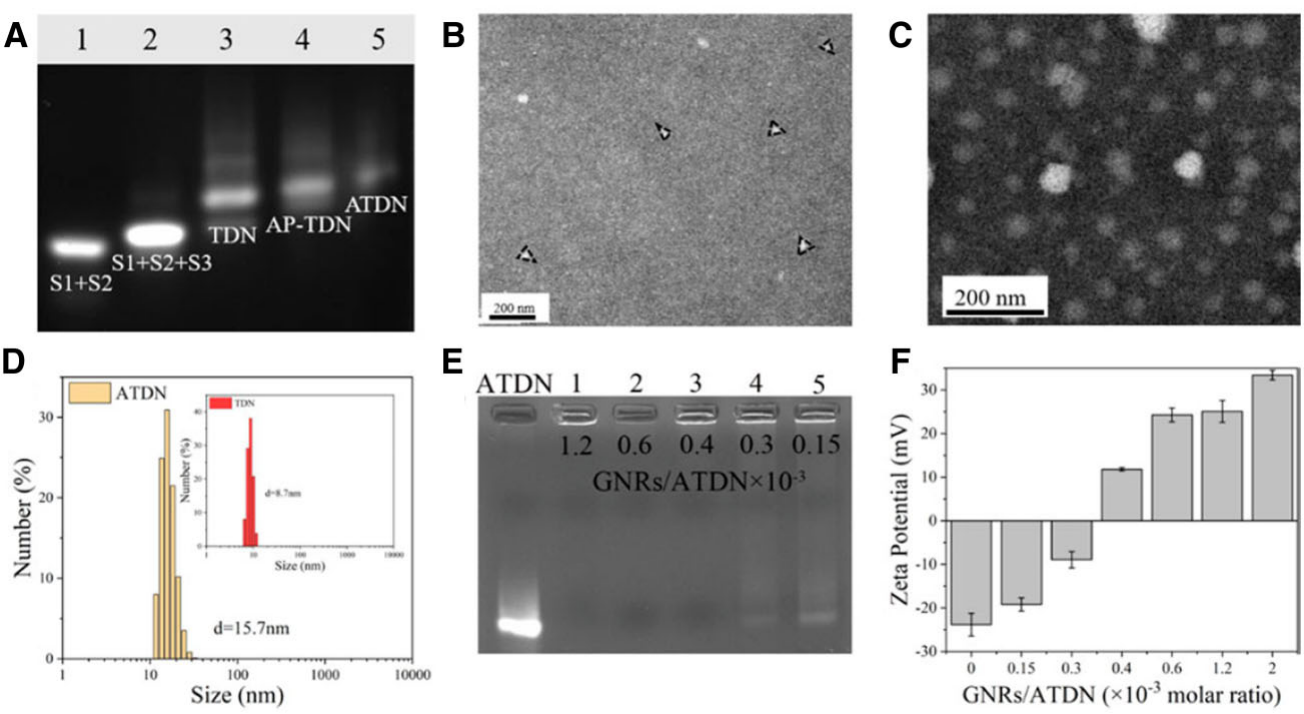
Gel retardation analysis of TDN and ATDN (**A**). TEM images of TDN and ATDN (**B** and **C**). The grain sizes of TDN and ATDN measured by DLS (**D**). Gel retardation analysis of GNRs@ATDN with different molar ratio of GNRs/ATDN (**E**). The zeta potentials of GNRs@ATDN with different molar ratio of GNRs/ATDN (**F**)

Tetrahedron DNA nanostructures (TDNs) are composed of four designed 55-mer oligonucleotides via base complementary pairing principle. A single strand and the other three complement for each other to form a tetrahedron. The 5′ and 3′ ends of each oligonucleotide can be ligated by ligase at the vertex of the TDN, and used as a functionalization site. Aptamer AS1411 conjugated the 5′ end of S2, S3 and S4 to receive aptamer-modified ssDNA (AS2, AS3, AS4). AP-TDN (containing an aptamer) and ATDN (containing three aptamers) were assembled with the same method. AGE assay was conducted to verify the formation of TDN and the aptamer-modified TDN. As demonstrated in Fig. 4A, TDN migrated tardier than other DNA combinations (S1 + S2, S1 + S2 + S3) attributing to the higher molecular weights. Lane 3 showed the synthetic TDNs. Lanes 4 and 5 represented the AP-TDN and ATDN, respectively. As the number of aptamers in TDNs increased, the bands showed slower migration rate.

The sizes of the TDN and aptamer-modified TDN were verified using DLS. Figure 4D showed that the hydrodynamic diameters of the DNA nanostructures increased with the amounts of aptamers. The particle size of TDN was 8.7 nm and that of ATDN increased to 15.7 nm. The morphology of the DNA nanostructures was demonstrated by TEM (Fig. 4B and C). The shapes of TDN and ATDN were approximately triangular, and the size of ATDN was slightly larger than that of TDN, which was in accordance with the results measured by DLS.

### Drug-loaded ATDN

TDN and ATDN were mixed with DOX to prepare drug-loaded nanoparticles. Through the conversion of the fluorescence intensity, the concentration of DOX in supernatant after centrifugation declined with increasing ATDN concentrations, displaying the successful encapsulation of DOX in ATDN (Supplementary Fig. S2B). As calculated by UV absorption (Supplementary Fig. S2A), 314 DOX molecules were intercalated into each ATDN, and the DLC (wt%) of DOX was 64.8%, which was much higher than that of polymer nanostructures [53].

### Characterization of GNRs@ATDN-DOX

Cationic polymers coated on GNRs could protect tetrahedron DNA from degradation and transport into cells. The ratio of cationic polymer to DNA affects the compression ability to DNA nanoparticles and endocytosis of nanomedicines. The GNRs-PEI compacted ATDN was attested by AGE at GNRs/ATDN × 10^−3^ molar ratios ranging from 0.15 to 1.2. Figure 4E showed the electrophoretic band of ATDN and the GNRs-PEI retarded ATDN with various GNRs/ATDN ratios (Lane 1–5). Once the molar ratio was higher than 0.4 × 10^−3^, ATDN band on the gel no longer migrated, which demonstrated that the mobility of ATDN was completely retarded and ATDN was completely compacted with GNRs-PEI. The zeta potentials of GNRs@ATDN-DOX nanoparticles with various GNRs/ATDN molar ratios were measured (Fig. 4F). As the molar ratio (GNRs/ATDN × 10^−3^) increased from 0.15 to 1.2, the zeta potential rose from –23.8 mV to +25.1 mV. At a higher molar ratio of 2.0, the zeta potential of GNRs@ATDN reached +33.4 mV. TEM result suggested that GNRs@ATDN-DOX maintained good dispersion of GNRs (Supplementary Fig. S4A). Typical UV–vis absorption of DOX at 480 nm was hardly observed in GNRs@ATDN-DOX (Supplementary Fig. S4B) suspension owing to the intrinsic absorption of GNRs at this wavelength and the intercalation interactions into DNA [54]. The DOX content was determined by measuring the absorbance of supernatant to be 64.76%, comparable to that in ATDN.

### Tumor targeting of aptamer AS1411

**Figure 5.**
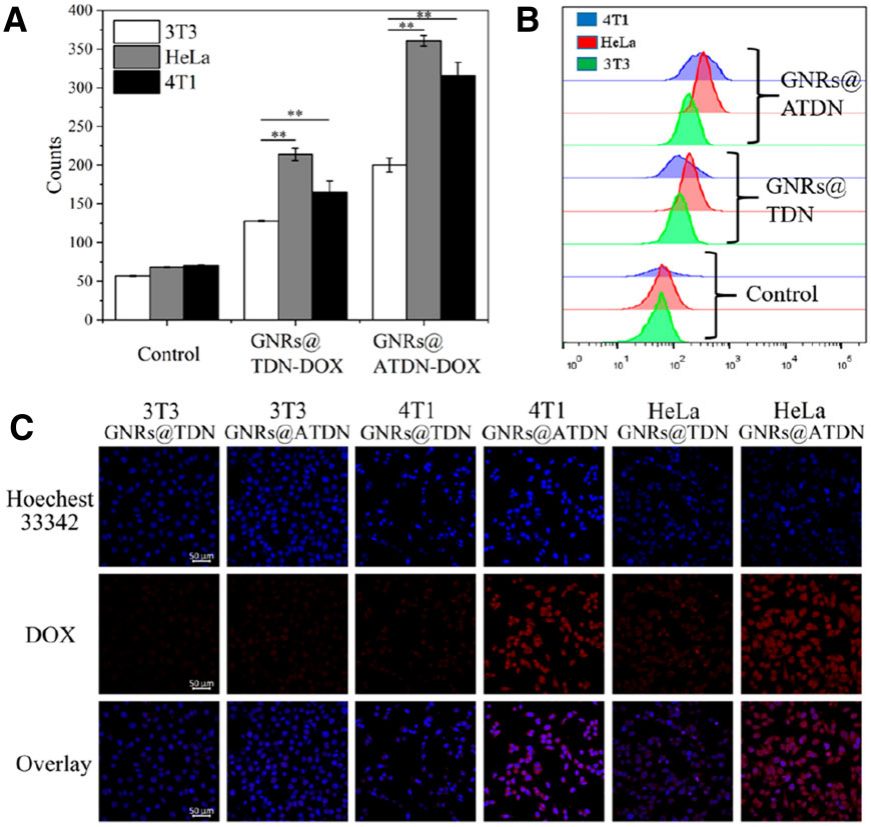
Cellular uptake of GNRs@ATDN-DOX and GNRs@TDN-DOX in 3T3, HeLa and 4T1 cells tested by flow cytometry (**A** and B) and CLSM (**C**). The results were expressed as mean ± SD (*n *=* *3, *P <* *0.05, **P <* *0.01)

Aptamer AS1411 is a guanine-rich oligonucleotide containing 26 bases, it forms stable G-quadruplex dimers and binds with highly expressed nucleolin on the cytomembrane of tumor cells with high affinity and specificity to interfere DNA replication through nucleolin shuttle, thus to inhibit cell proliferation [55]. The tumor targeting of AS1411 was investigated. CLSM and flow cytometry were applied to study cellular uptake of GNRs@ATDN-DOX and GNRs@TDN-DOX in HeLa, 4T1 and 3T3 cells. As shown in Fig. 5C, the fluorescence intensity of GNRs@ATDN-DOX in HeLa and 4T1 cells was apparently higher than that of 3T3 cells. The fluorescence intensity of GNRs@TDN-DOX was nearly the same in the three cells. AS1411-modified nanoparticles exhibited targeting effect to HeLa and 4T1 tumor cells. From the results of flow cytometry (Fig. 5A and B), HeLa cells and 4T1 cells showed apparently higher than 3T3 cells in the cellular uptake ability of GNRs@ATDN-DOX. The cellular uptake of GNRs@ATDN-DOX by 3T3, HeLa and 4T1 cells was 1.58, 1.69 and 1.92 times as those of GNRs@TDN-DOX group, clearly confirming that AS1411-modified nanocarriers showed significant tumor cells targeting ability.

### *In vitro* cytotoxicity

**Figure 6.**
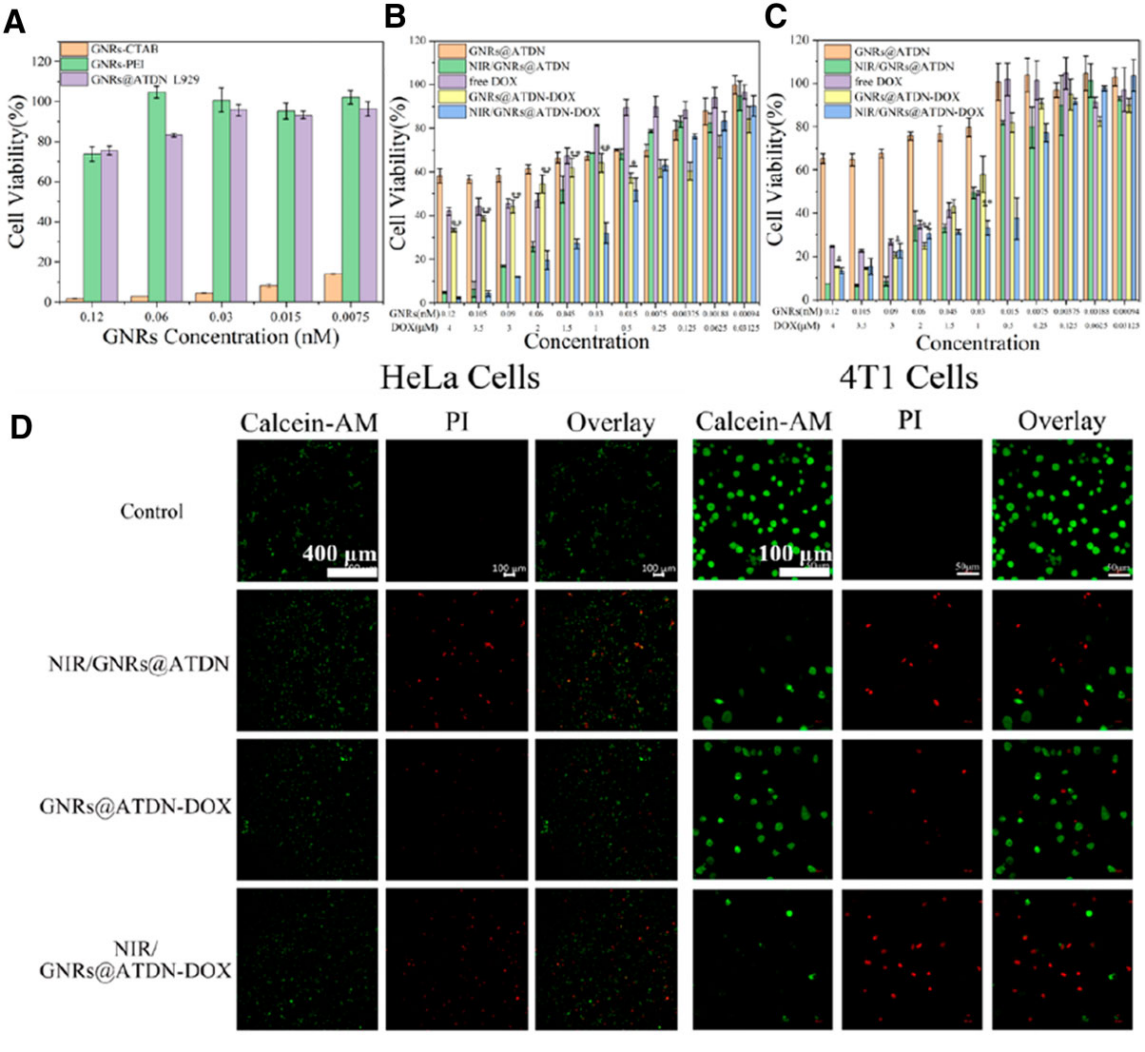
*In vitro* cytotoxicity of GNRs-CTAB, GNRs-PEI and blank GNRs@ATDN in 4T1 and L929 cells (**A**). The cytotoxicity of free DOX, GNRs@ATDN, NIR/GNRs@ATDN, GNRs@ATDN-DOX and NIR/GNRs@ATDN-DOX in HeLa and 4T1 cells (**B** and **C**). LIVE/DEAD viability/cytotoxicity assay of NIR/GNRs@ATDN, GNRs@ATDN-DOX and NIR/GNRs@ATDN-DOX in HeLa and 4T1 cells (**D**). The results were expressed as mean ± SD (*n *=* *5, *P <* *0.05, **P <* *0.01)

The cytotoxicity of GNRs-CTAB, GNRs-PEI and blank GNRs@ATDN nanoparticles against 4T1 breast cancer cells and L929 fibroblasts was calculated using an MTT assay. As displayed in Fig. 6A, the GNRs coated with CTAB killed almost all cells even at very low concentrations. When PEI replaced CTAB layer, the cell viability was improved, certain cytotoxicity appeared in the nanoparticles with high concentrations, which was attribute to the strong positive charge of 25 kDa PEI.

The inhibition effects of free DOX, GNRs@ATDN, NIR/GNRs@ATDN, GNRs@ATDN-DOX and NIR/GNRs@ATDN-DOX to the proliferation of HeLa and 4T1 cells were estimated *in vitro*. As shown in Fig. 6B and C, the GNRs@ATDN group showed low toxicity even at high concentration. The cytotoxicity of single photothermal group (NIR/GNRs@ATDN) was slightly stronger than that of sole chemotherapy group (GNRs@ATDN-DOX). The anti-tumor effect of GNRs@ATDN-DOX group was stronger than that of free DOX group as GNRs@ATDN-DOX group possessed both targeting and lysosomal escape functions. The combination index (CI) values of NIR/GNRs@ATDN-DOX was calculated to be 0.37 and 0.55 in HeLa and 4T1 cells, respectively, indicating the synergistic anticancer effect. The anti-cancer effect of NIR/GNRs@ATDN-DOX group was the best because it combined targeting delivery, lysosomal escape, and efficient photothermal effect to induce cell apoptosis. In addition, localized heat could also facilitate the cellular uptake of nanoparticles [56], decrease surface area of GNRs [57] and denature DNA double helix [58], which enabled enhanced cellular uptake and DOX release in cancer cells. The anti-tumor results were further verified by LIVE/DEAD viability/cytotoxicity assay (Fig. 6D). The number of viable cells (green fluorescence) in NIR/GNRs@ATDN and GNRs@ATDN-DOX groups was significantly higher than that in NIR/GNRs@ATDN-DOX group, and the NIR/GNRs@ATDN-DOX treatment caused most cells death (red fluorescence). The GNRs@ATDN-DOX nanoparticles with 808 nm laser irradiation generated heat to kill tumor cells together with chemotherapeutics.

### Lysosome escape

**Figure 7.**
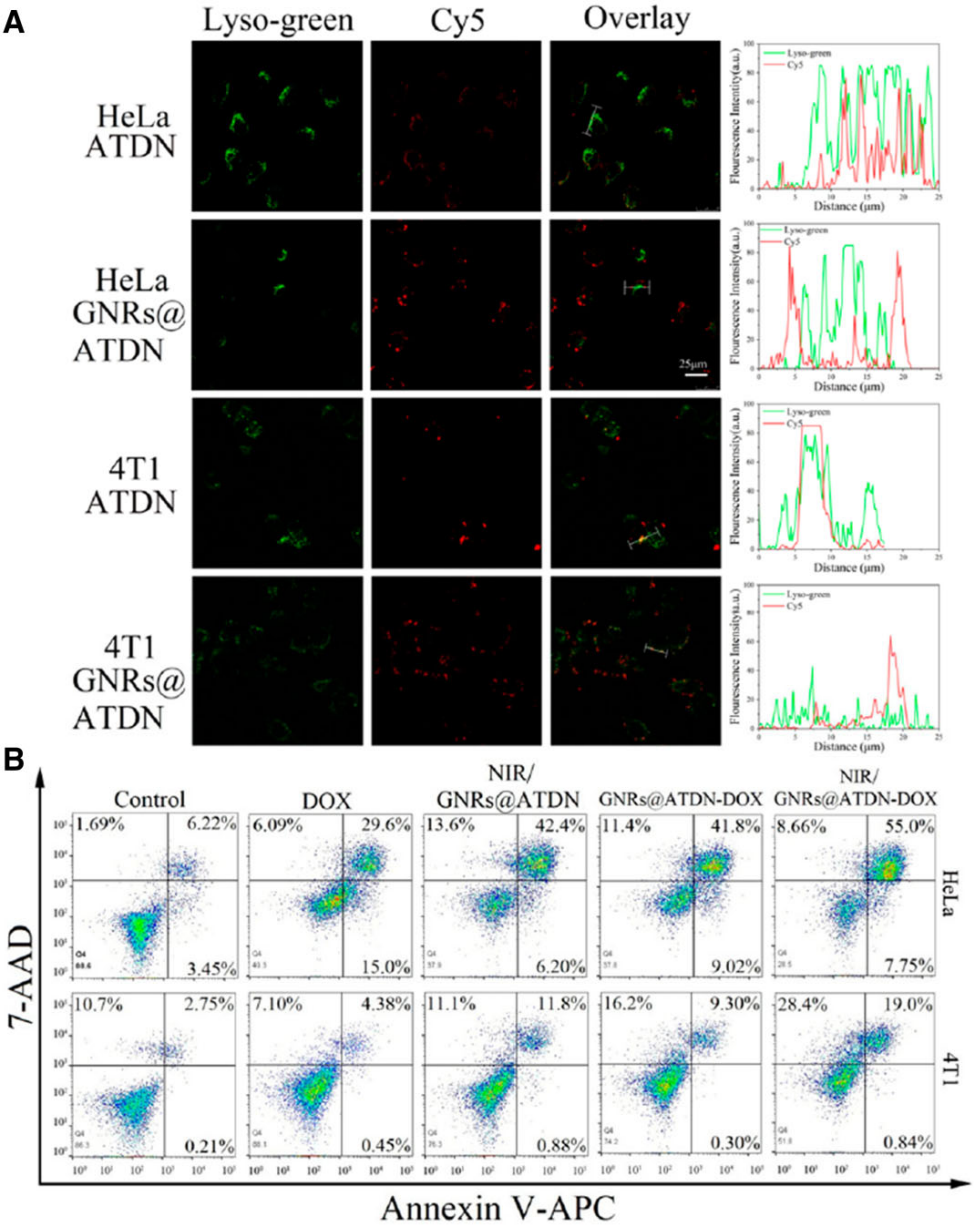
Lysosome escape ability of ATDN and GNRs@ATDN in HeLa and 4T1 cells (A). Cell apoptosis of HeLa and 4T1 cells treated by free DOX, NIR/GNRs@ATDN, GNRs@ATDN-DOX and NIR/GNRs@ATDN-DOX (**B**).

Lysosomes contain a variety of hydrolases to decompose bio-macromolecules which contain proteins, nucleic acids and polysaccharides in cells. Therefore, lysosome escape is the key factor for nanomedicines to effectively kill tumor cells. The large number of amino groups in PEI could capture H^+^ in low pH value environment in lysosomes. In order to maintain the charge balance, the influx of Cl^–^ and water from cytoplasm leads to osmotic swelling and rupture lysosomes to release nanoparticles. The lysosome escape of the Cy5-labeled DNA nanostructures was investigated by CLSM. As illustrated in Fig. 7A, the fluorescence co-location of red (ATDN labeled by Cy5) and green fluorescence (Lyso-trackers^®^ Green) was analyzed, demonstrating the effective internalization of ATDN and GNRs@ATDN. After a period of incubation, the yellow fluorescence overlapped of green and red fluorescence was discovered in both cells treated with ATDN, revealing the entrapment of ATDN in endosomes. In contrast, the red fluorescence of Cy5 did not overlap with the green fluorescence of lysosomes in the GNRs@ATDN-treated group, it indicated that owing to the proton sponge effect of PEI, DNA nanocarriers successfully escaped from lysosomes, which was beneficial to help drug entering nucleus to promote anti-tumor effect.

### Cell apoptosis assay

Cell apoptosis induced by chemotherapeutic agent (DOX) and photothermal GNRs was detected via Annexin-V/7-AAD commercial kit. HeLa and 4T1 cells were cultivated with free DOX, NIR/GNRs@ATDN, GNRs@ATDN-DOX and NIR/GNRs@ATDN-DOX for 12 h with the same DOX concentration of 2 μM. The cells were illuminated by NIR (808 nm, 1 W cm^−2^) for 5 min in NIR/GNRs@ATDN- and NIR/GNRs@ATDN-DOX-treated groups. The results of flow cytometry-based apoptosis assay (Fig. 7B) showed that apoptotic rate of GNRs@ATDN-DOX to HeLa cells was evidently stronger than that of free DOX, and the proportion of late apoptosis and necrosis increased from 29.6% to 41.8%. The apoptosis ratio of NIR/GNRs@ATDN group (NIR only) and GNRs@ATDN-DOX (chemotherapy only) group was nearly the same, and the apoptosis ratio of synergistic photothermal–chemotherapy group was the highest as 62.75%. Similar to HeLa cells, 4T1 cells also exhibited the alike apoptosis trend. The GNRs@ATDN-DOX group caused 9.30% of late apoptosis, which was stronger than 4.38% of the free DOX group, indicating the significant enhancement of nanofabricated formulations. The proportion of apoptosis caused by NIR/GNRs@ATDN-DOX group (19.84%) was higher than that of sole photothermal or chemotherapy group, which further confirmed that the combination therapy showed better anticancer ability than single therapy.

## Conclusions

In summary, GNRs and ATDN nanocomposites with lysosome escape were developed for targeting chemo-photothermal combination therapy. The encapsulation of DOX in ATDN exhibited high drug-loading capacity. The aptamer AS1411-modified DNA tetrahedron accomplished tumor cells targeting to enhance the precision of drug delivery system. The PEI layer on GNRs compacted ATDN-DOX to endow lysosome escape ability. GNRs@ATDN-DOX nanoparticles under NIR irradiation induced severe apoptosis and necrosis to cancer cells, demonstrating better synergistic effect with the collaborative of photothermal and chemotherapy. The GNR/ATDN-based nanocomposite delivery platform exhibits a broad prospect for efficient tumor therapy.

## Supplementary data

Supplementary data are available at *REGBIO* online.

## Funding

This work was supported by the National Natural Science Foundation of China (51873121).

*Conflicts of interest statement*. None declared.

## Supplementary Material

rbac032_Supplementary_Data
