# Supplementary material for: Gold nanorods/tetrahedral DNA composites for chemo-photothermal therapy
Source: Regen Biomater. 2022 May 4;9:rbac032. doi: 10.1093/rb/rbac032 (PMC9163824; doi:10.1093/rb/rbac032)
Supplement: rbac032_Supplementary_Data [file rbac032_supplementary_data.docx]

Supporting Information of

Gold Nanorods/Tetrahedral DNA Composites for Chemo-photothermal Therapy

Ziyun He^1^, Qiusheng Wang^1^, Nan Zhang^1^, Jianqin Yan^2^, Li Li^1^, Jun Cao^1^, and Bin He^1*^

^1^ National Engineering Research Center for Biomaterials, College of Biomedical Engineering, Sichuan University, Chengdu 610064, China

^2^ Department of Pharmaceutics, School of Pharmacy, Qingdao University, Qingdao 266073, China.

**Materials.** Hoechst 33342, LIVE/DEAD viability/cytotoxicity kit, Annexin-V/ 7-AAD apoptosis kit and Lyso-tracker Green were purchased from Beyotime Institute of Biotechnology (Shanghai, China). 3-(4, 5-Dimethylthiazol-2-yr)-2, 5-diphenyltetrazolium bromide (MTT) and lipoic acid (LA) were obtained from Aladdin (Shanghai, China). Cervix cancer (HeLa) cells, mice breast cancer (4T1) cells, L929 fibroblast cells and NIH/3T3 cells were obtained from the Chinese Academy of Science Cell Bank for Type Culture Collection (Shanghai, China). Dulbecco’s Modified Eagle’s Medium (DMEM)， Roswell Park Memorial Institute 1640 medium (RPMI 1640), fetal bovine serum (FBS) and penicillin-streptomycin solution were purchased from Life Technologies Co. (Gibco, USA).

**Cell culture.** HeLa, 4T1, 3T3 and L929 cells were cultured in DMEM with 10% fetal bovine serum (FBS), 100 U mL^−1^ penicillin and streptomycin in a 95% humidity atmosphere containing 5% CO_2_ at 37 ℃.

**Table S1.** DNA oligonucleotides used in TDN, ATDN and Cy5-labeled ATDN.

| ssDNA | Sequence (5’–3’) |
| --- | --- |
| S1 | ACATTCCTAAGTCTGAAACATTACAGCTTGCTACACGAGAAGAGCCGCCATAGTA |
| S2 | TATCACCAGGCAGTTGACAGTGTAGCAAGCTGTAATAGATGCGAGGGTCCAATAC |
| S3 | TCAACTGCCTGGTGATAAAACGACACTACGTGGGAATCTACTATGGCGGCTCTTC |
| S4 | TTCAGACTTAGGAATGTGCTTCCCACGTAGTGTCGTTTGTATTGGACCCTCGCAT |
| AS2 | GGTGGTGGTGGTTGTGGTGGTGGTGGTTTTTTTTATCACCAGGCAGTTGACAGTGTAGCAAGCTGTAATAGATGCGAGGGTCCAATAC |
| AS3 | GGTGGTGGTGGTTGTGGTGGTGGTGGTTTTTTTTCAACTGCCTGGTGATAAAACGACACTACGTGGGAATCTACTATGGCGGCTCTTC |
| AS4 | GGTGGTGGTGGTTGTGGTGGTGGTGGTTTTTTTTTCAGACTTAGGAATGTGCTTCCCACGTAGTGTCGTTTGTATTGGACCCTCGCAT |
| Cy5-S1 | Cy5-ACATTCCTAAGTCTGAAACATTACAGCTTGCTACACGAGAAGAGCCGCCATAGTA |


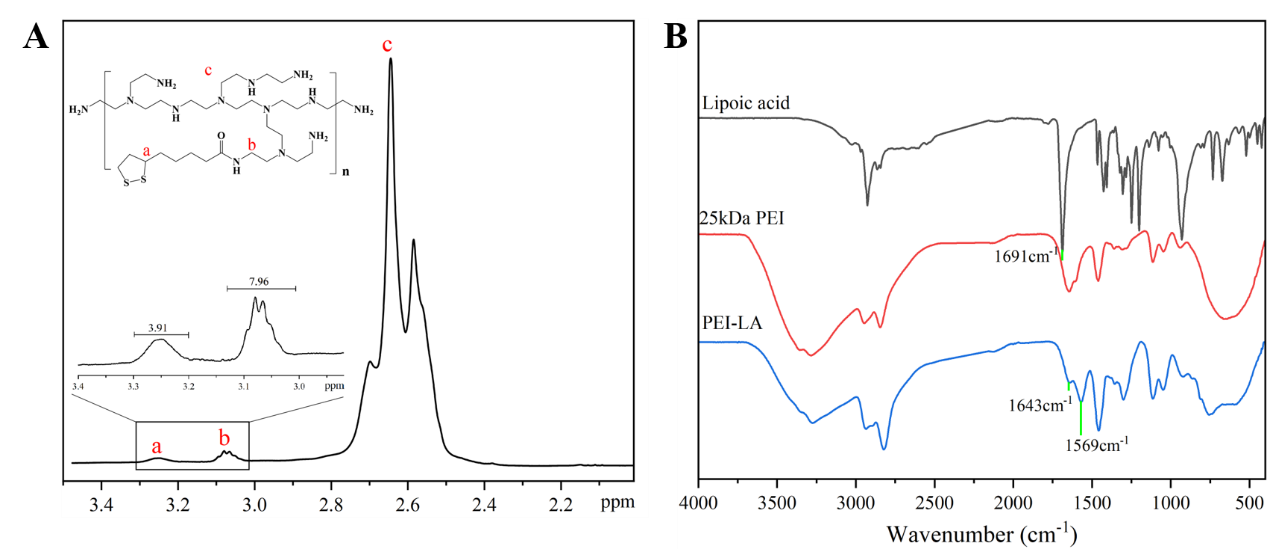


**Figure S1.** (A) ^1^H NMR spectrum of PEI-LA in D_2_O. (B) FT-IR spectra of LA, PEI, and PEI-LA.


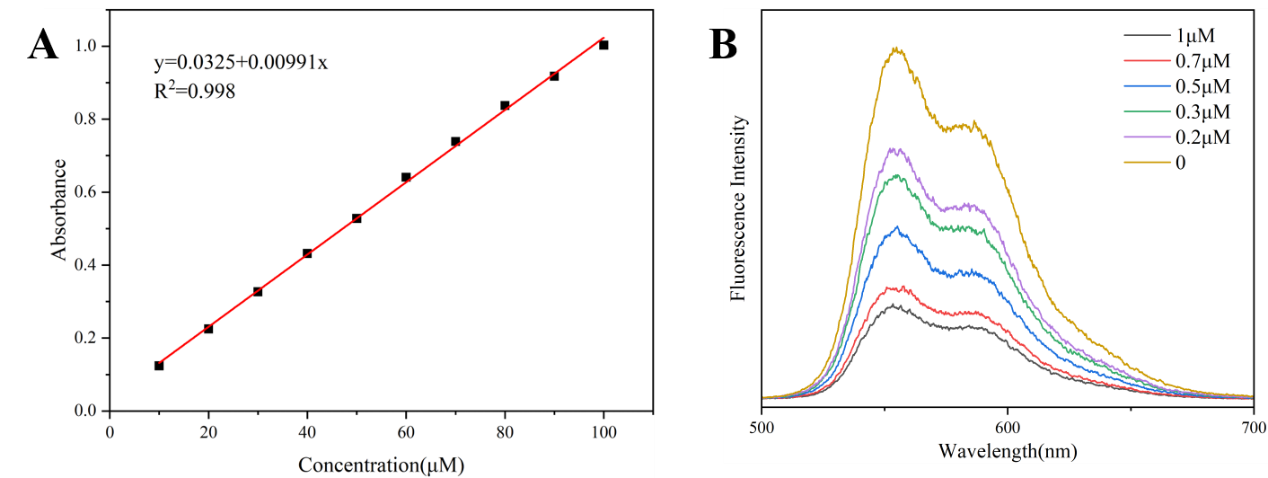


**Figure S2.** (A) UV absorption standard curve of DOX. (B) Fluorescence intensity of different concentrations of ATDN incubated with DOX (500 μM).


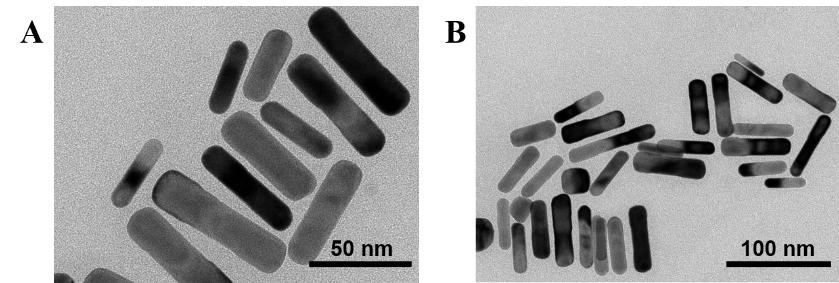


**Figure S3.** TEM image of GNRs-CTAB.


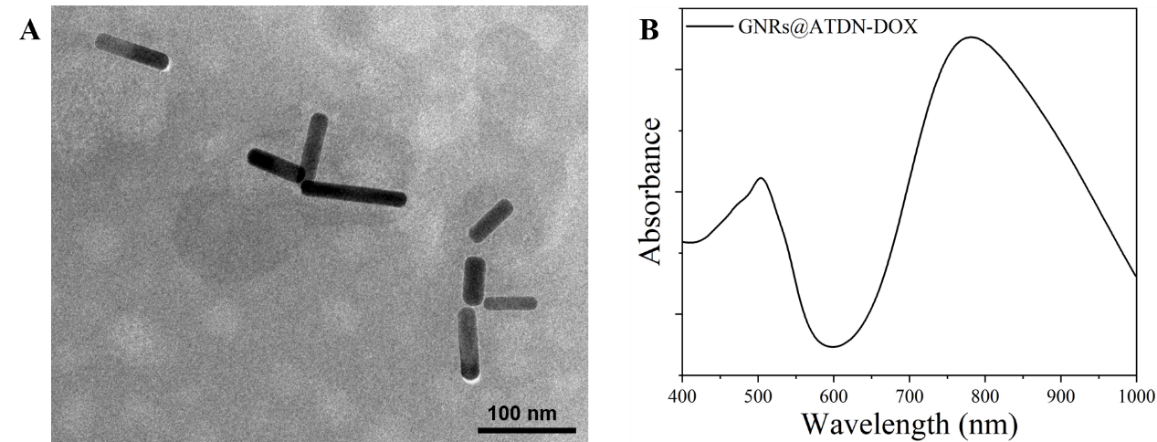


**Figure S4.** (A) TEM image of GNRs@ATDN-DOX. (B) UV-vis absorption of GNRs@ATDN-DOX.
